# Distribution and persistence of pleural penetrations by multi-walled carbon nanotubes

**DOI:** 10.1186/1743-8977-7-28

**Published:** 2010-10-04

**Authors:** Robert R Mercer, Ann F Hubbs, James F Scabilloni, Liying Wang, Lori A Battelli, Diane Schwegler-Berry, Vincent Castranova, Dale W Porter

**Affiliations:** 1 Pathology and Physiology Research Branch, HELD, NIOSH, Morgantown, WV, USA; 2 Department of Physiology and Pharmacology, West Virginia University, Morgantown, WV, USA

## Abstract

**Background:**

Multi-walled carbon nanotubes (MWCNT) are new manufactured nanomaterials with a wide spectrum of commercial applications. The durability and fiber-like dimensions (mean length 3.9 μm long × 49 nm diameter) of MWCNT suggest that these fibers may migrate to and have toxicity within the pleural region. To address whether the pleura received a significant and persistent exposure, C57BL/6J mice were exposed by pharyngeal aspiration to 10, 20, 40 and 80 μg MWCNT or vehicle and the distribution of MWCNT penetrations determined at 1, 7, 28 and 56 days after exposure. Following lung fixation and sectioning, morphometric methods were used to determine the distribution of MWCNT and the number of MWCNT fiber penetrations of three barriers: alveolar epithelium (alveolar penetrations), the alveolar epithelium immediately adjacent to the pleura (subpleural tissue), and visceral pleural surface (intrapleural space).

**Results:**

At 1 day 18%, 81.6% and 0.6% of the MWCNT lung burden was in the airway, the alveolar, and the subpleural regions, respectively. There was an initial, high density of penetrations into the subpleural tissue and the intrapleural space one day following aspiration which appeared to decrease due to clearance by alveolar macrophages and/or lymphatics by day 7. However, the density of penetrations increased to steady state levels in the subpleural tissue and intrapleural from day 28 - 56. At day 56 approximately 1 in every 400 fiber penetrations was in either the subpleural tissue or intrapleural space. Numerous penetrations into macrophages in the alveolar airspaces throughout the lungs were demonstrated at all times but are not included in the counts presented.

**Conclusions:**

The results document that MWCNT penetrations of alveolar macrophages, the alveolar wall, and visceral pleura are both frequent and sustained. In addition, the findings demonstrate the need to investigate the chronic toxicity of MWCNT at these sites.

## Background

Carbon nanotubes (CNT) are nanometer diameter tubes of pure carbon which are being developed and produced in mass quantities for a variety of applications such as strengthening of composite materials, ballistics fabrics, medical imaging, drug delivery and as a key component in lithium batteries. CNT production is estimated to reach into the millions of tons [1]. Many variations on the form, end configuration and number of concentric shells or tubes of the carbon atoms are actively being developed with single-walled carbon nanotubes (SWCNT) and multi-walled carbon nanotubes (MWCNT) being the two principal forms.

Due to the physical and chemical durability and fibrous shape of MWCNT, concern has been raised that MWCNT may exhibit potentially significant health hazards similar to asbestos[2]. A recent report demonstrates that intraperitoneal instillation of MWCNT in p53 +/- mice results in mesothelioma [3]. This study has been questioned due to the high dose of MWCNT (3 mg) injected into the abdomen[4]. However, a follow up preliminary report from the same group demonstrated mesothelioma also occurred after intraperitoneal injection of as little as 50 μg/mouse [5]. Abdominal and thoracic mesothelioma was also reported after intrascrotal injection of MWCNT into Fischer rats [6]. Poland et al [7] reported that intraperitoneal injection of 50 μg of long, but not short MWCNT, in mice resulted in inflammation and granulomatous lesions on the abdominal side of the diaphragm at 1-2 weeks post-exposure. In contrast, Muller et al. [8] reported no mesothelioma 2 years after intraperitoneal injection of MWCNT. However, the MWCNT sample used in this study consisted of very short fibers (< 1 μm), which would have been predicted by Poland and coworkers to exhibit low bioactivity in that assay. Although the data above indicate that MWCNT can cause mesothelioma after intraperitoneal instillation, as does asbestos, data are required that MWCNT actually come in contact with the mesothelial cells lining the lung after pulmonary exposure. A review by Donaldson et al. [9] argues that migration into the intrapleural space is common for particles deposited in the distal lung. However, risk assessment requires evidence that MWCNT not only can reach the intrapleural space but quantification of the dose-dependence and time course of such migration.

The multiple concentric walls of carbon in MWCNT are substantially more rigid than SWCNT and are found to penetrate and/or pass through cells and membranes in the exposed lungs. We have previously reported the extensive degree to which lungs respond to MWCNT, including the rapid development of pulmonary fibrosis by 7 days post-exposure and the formation of granulomatous lesions [10,11]. In that study, MWCNT were observed to reach the pleura after pulmonary exposure. This result is supported by Ryman-Rasmussen et al. [12] who reported that MWCNT can reach the subpleural tissue after inhalation. In order to conduct an evaluation of this potential hazard, further study is necessary. Determination of the proportion of the lung burden which is transported to the pleura, the time-course of the transport, and finally the dose-response of tissues in the pleura which are exposed to the transported MWCNT, should be determined. The present study was designed to extend the initial observations by measurement of MWCNT migration to the subpleural tissue and intrapleural space at different lung burdens and determination of the time course of that transport.

## Results

**Figure 1:**
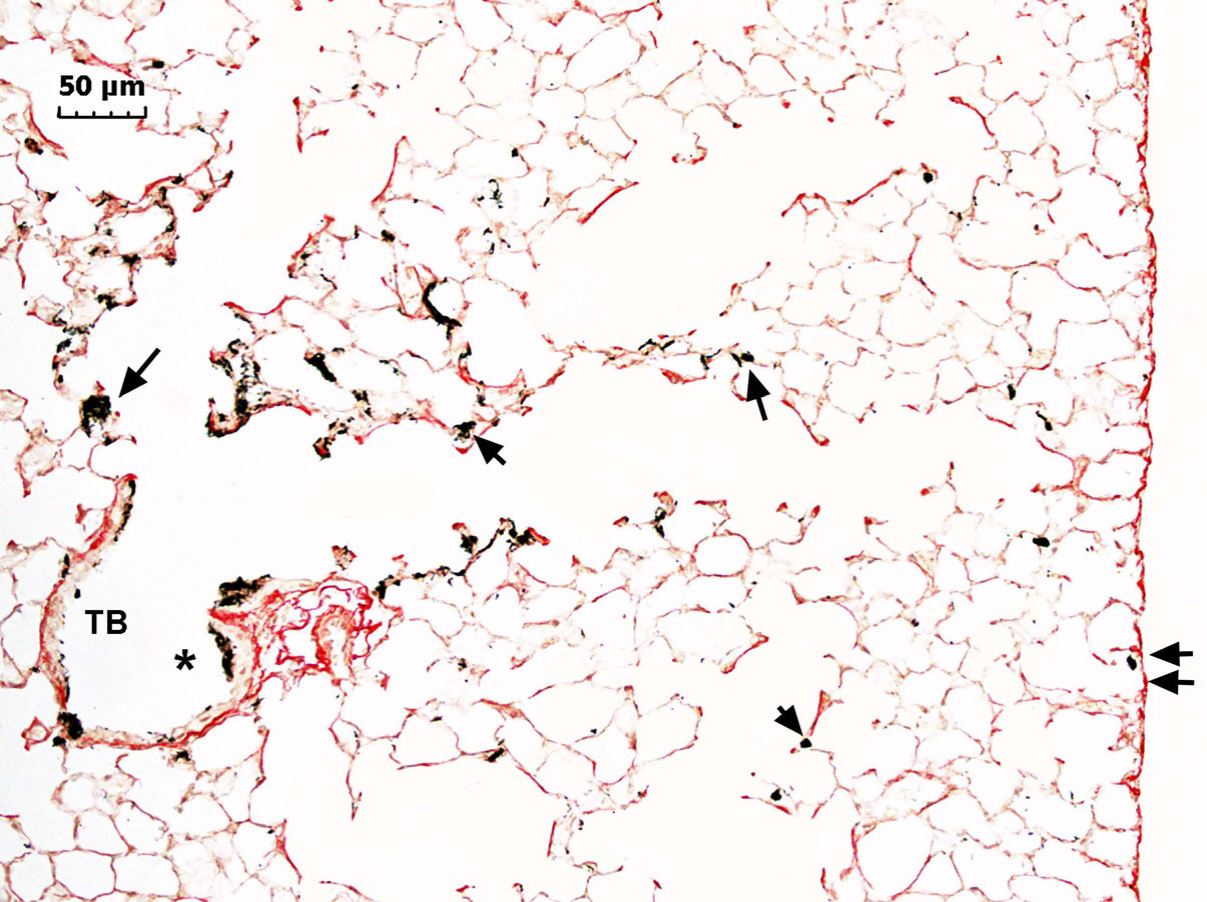
**Light micrograph of MWCNT deposition in alveolar region of lungs**. Sirius Red stained micrograph showing the general deposition pattern of MWCNT (arrows) one day after aspiration. A deposit of MWCNT on the epithelium of the terminal bronchiole (TB) near the transition between the airways and the alveolar region is indicated by the asterisk. Smaller deposits near the subpleural tissue region are indicated by double arrows. (dose 80 μg).

The light microscopic section of Figure 1 shows a representative cross-section from terminal bronchiole out to the pleural surface of the lungs 1 day after aspiration of a 80 μg dose. As illustrated in the micrograph, MWCNT were deposited throughout the alveolar region of the lungs with the highest concentrations visible in the alveoli immediately proximal to the terminal bronchiole.

**Figure 2:**
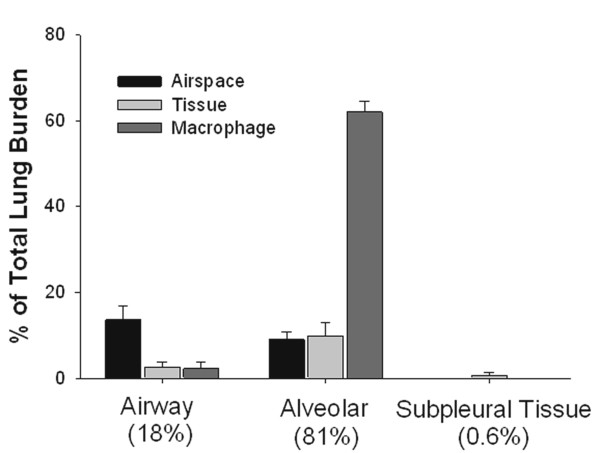
**Morphometric determination of the initial lung distribution of MWCNT 1 day post aspiration**. Results show the distribution of MWCNT fiber burden in airways, alveolar and subpleural tissue regions of the lungs one day after aspiration. As shown by the different shades of bars, MWCNT fiber burden in the airways and alveolar regions were further subdivided into the airspaces of the region, tissue of the region and macrophages. Results are expressed as a percentage of the total lung burden. (Mean ± SE, N = 7).

The lung burden distribution of MWCNT at 1 day post-aspiration (80 μg) is shown in Figure 2. The results demonstrate the prominent role of alveolar macrophages. The majority of the lung burden was deposited in the alveolar region with alveolar macrophages receiving 62% of the total dose. The airways and alveolar regions accounted for 18 and 81% of the lung burden, respectively. MWCNT in the visceral pleura region (subpleural tissue and intrapleural space) accounted for approximately 0.6% of the total lung burden.

**Figure 3:**
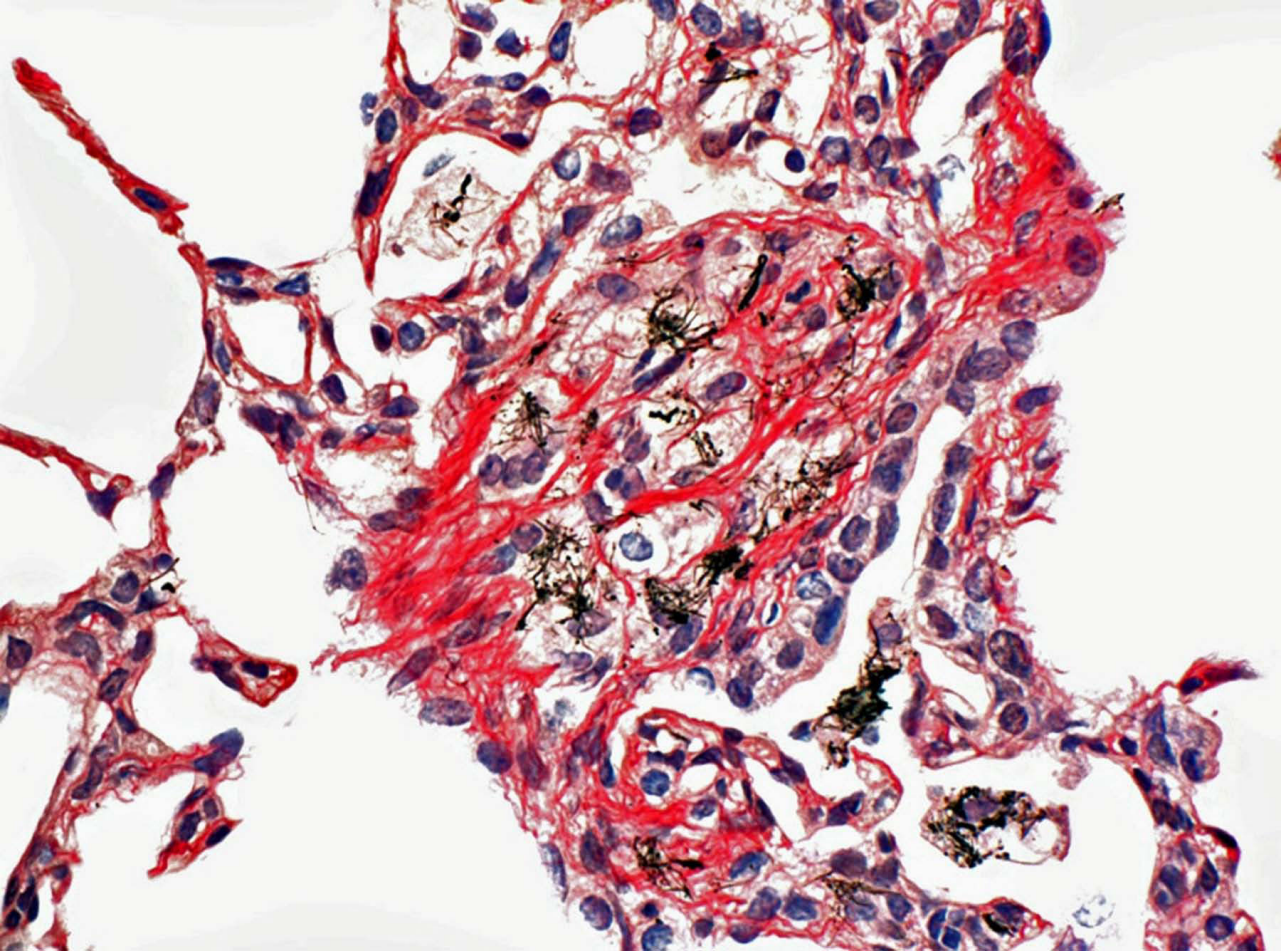
**Light micrograph of fibrotic granulomas 56 days following MWCNT**. Micrograph illustrates the extensive collagen network developed within granulomas that developed following a single 20 μg dose of MWCNT. Section stained with Sirius Red show collagen fibers in red.

A representative image of the granulomatous lesions formed by MWCNT 56 days after aspiration of a 20 μg dose is shown in Figure 3. Collagen fibers in this section are stained in red. The section illustrates the highly fibrotic nature of the lung response to well dispersed MWCNT. These fibrotic nodules were rare in number compared to similar lesions previously reported for poorly dispersed SWCNT [13-15]. These earlier studies with SWCNT did not use dispersants in the media, resulting in many densely packed, 15-20 micron diameter clumps of SWCNT in the lungs immediately following exposure. The lung response to such large, "foreign" bodies is to wall-off the material with epitheloid macrophages resulting in the prominent granulomas illustrated in studies that rely solely on mechanical means to disperse SWCNT [13-15]. Use of a well-dispersed preparation of SWCNT has been shown to decrease granuloma formation and enhance the diffuse interstitial fibrotic response from the sub-micron SWCNT [16].

**Figure 4:**
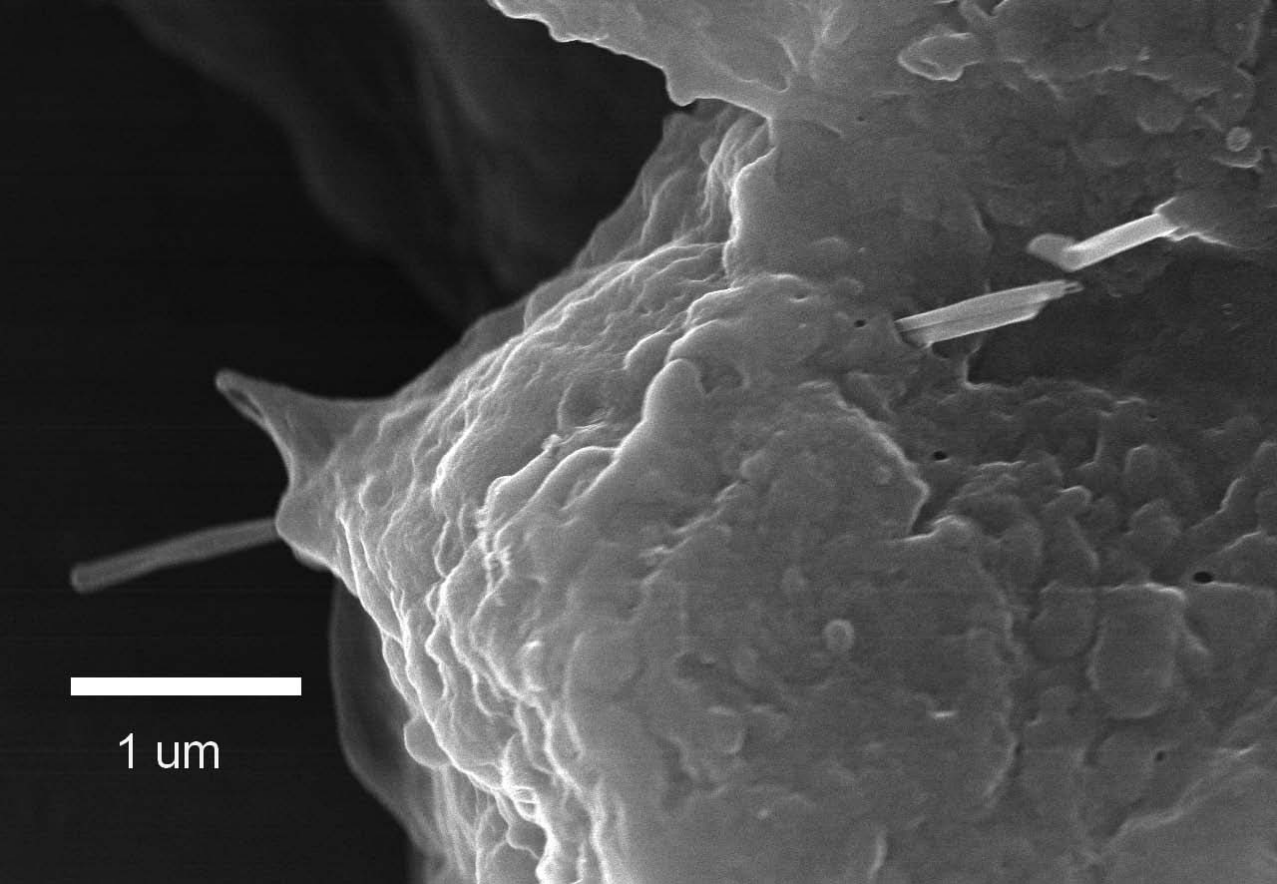
**FESEM of MWCNT penetration of alveolar epithelial cells**. As shown in this micrograph, MWCNT were rapidly incorporated into the alveolar epithelium. Micrograph shows two MWCNT passing through an alveolar epithelial cell 1 day after aspiration (80 ug dose). The two fibers are running together on the right side of the cell and appear to separate within the cell as they pass out on the left side of the cell. Another MWCNT penetrates the epithelium in the upper right of micrograph.

The FESEM micrograph of Figure 4 demonstrates the direct penetration of an alveolar Type I epithelial cell by multiple MWCNT one day after an 80 μg dose. The penetration process was rapid and observed to be most frequent for alveolar macrophages followed by alveolar Type I epithelial cells and to a lesser extent by alveolar interstitial cells which were typically penetrated by a fiber as it passed through an adjacent epithelial cell. The FESEM observations confirmed the prior light microscopy based observations that MWCNT frequently extend from the cell surface through cell nuclei as well as other organelles and were not confined to phagolysosomes [10]. Alveolar Type II epithelial cells, which account for 2% of the normal epithelial surface [17], were rarely found to have penetrations by MWCNT. MWCNT could be observed in the mucous lining layers above airway epithelial cells and in airway macrophages contained in the cilia-mucous lining layer of the airways but penetrations by MWCNT were rarely observed.

**Figure 5:**
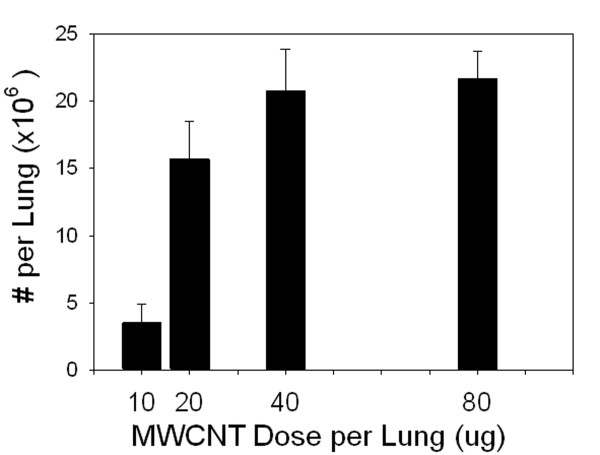
**Morphometric determination of the number of alveolar epithelial penetrations versus dose 56 days after aspiration**. The number of MWCNT penetrations per lung versus aspiration dose of MWCNT demonstrated a sigmoidal dose-response with an ED_50 _of 15.3 μg. (Mean ± SE, N = 8).

Based on the morphometric analysis, there were substantial number of alveolar epithelial cells penetrations by MWCNT (Figure 5). A normal mouse lung contains 11 million alveolar Type I epithelial cells [17] and there were 15 million MWCNT penetrations observed at the 20 μg dose. Thus at this dose, on average, every Type I epithelial cell would have approximately one and half MWCNT penetration. We did not determine the number of alveolar macrophage penetrations as the number and degree of overlap by multiple penetrations were too great to make accurate counting of individual penetrations practical. There are approximately 3 million alveolar macrophages in the normal mouse lung [17] and the alveolar macrophage lung distribution of MWCNT was approximately 6 times that of the tissue (Figure 2). Thus, there are one third the cell number receiving 6 times the dose. This which would indicate that the alveolar macrophage penetrations might be 18-fold higher than the alveolar epithelial dose per cell or 27 MWCNT penetrations per alveolar macrophage at the 20 μg dose. The number of MWCNT penetrations per lung versus aspiration dose of MWCNT demonstrated a sigmoidal dose-response with an ED50 of 15.3 μg.

**Figure 6:**
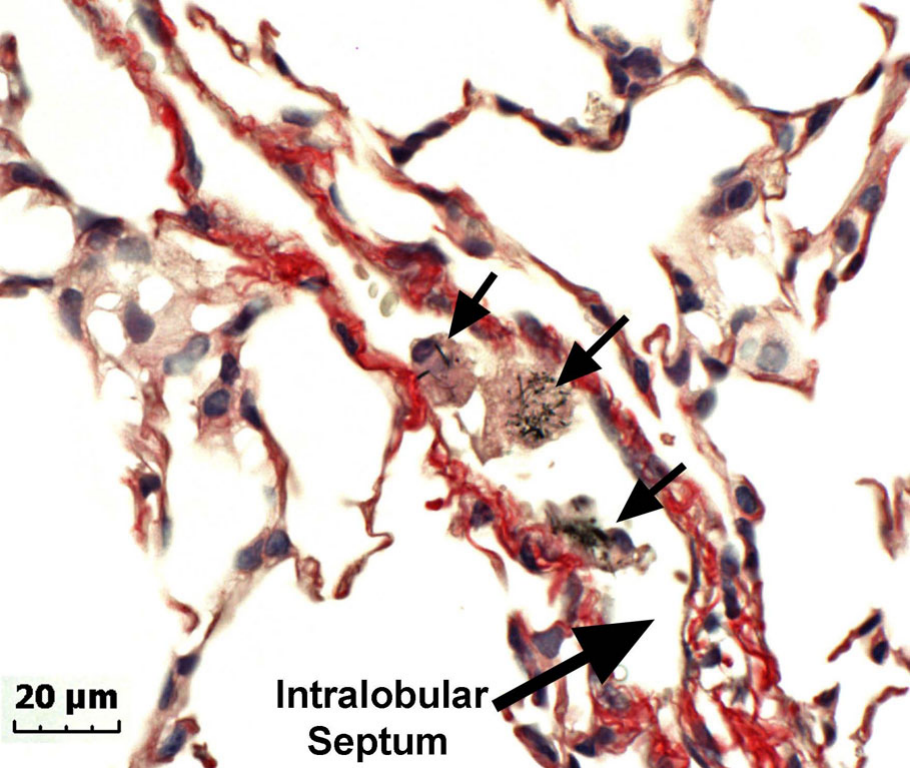
**Photomicrograph of lung section showing an intralobular septum**. This micrograph shows several MWCNT loaded macrophages (arrows) in the intralobular septum at 56 days post aspiration (40 μg dose).

Lung fixation in-situ followed by removal of the lungs from the chest cavity was used to obtain lung tissue in this study. This method does not preserve the contents of the pleural space. In order to preserve the pleural space contents special methods, such as interpleural injection of agarose, are required [18]. However, intralobular septa are relatively undisturbed by the fixation technique used in our study and intralobular septa were found to contain MWCNT within macrophages as shown by the representative photomicrograph (Figure 6) which was taken at 56 days post-aspiration of a 40 μg dose. The presence of MWCNT loaded macrophages in intralobular septa indicates significant MWCNT transport to the pleural space.

**Figure 7:**
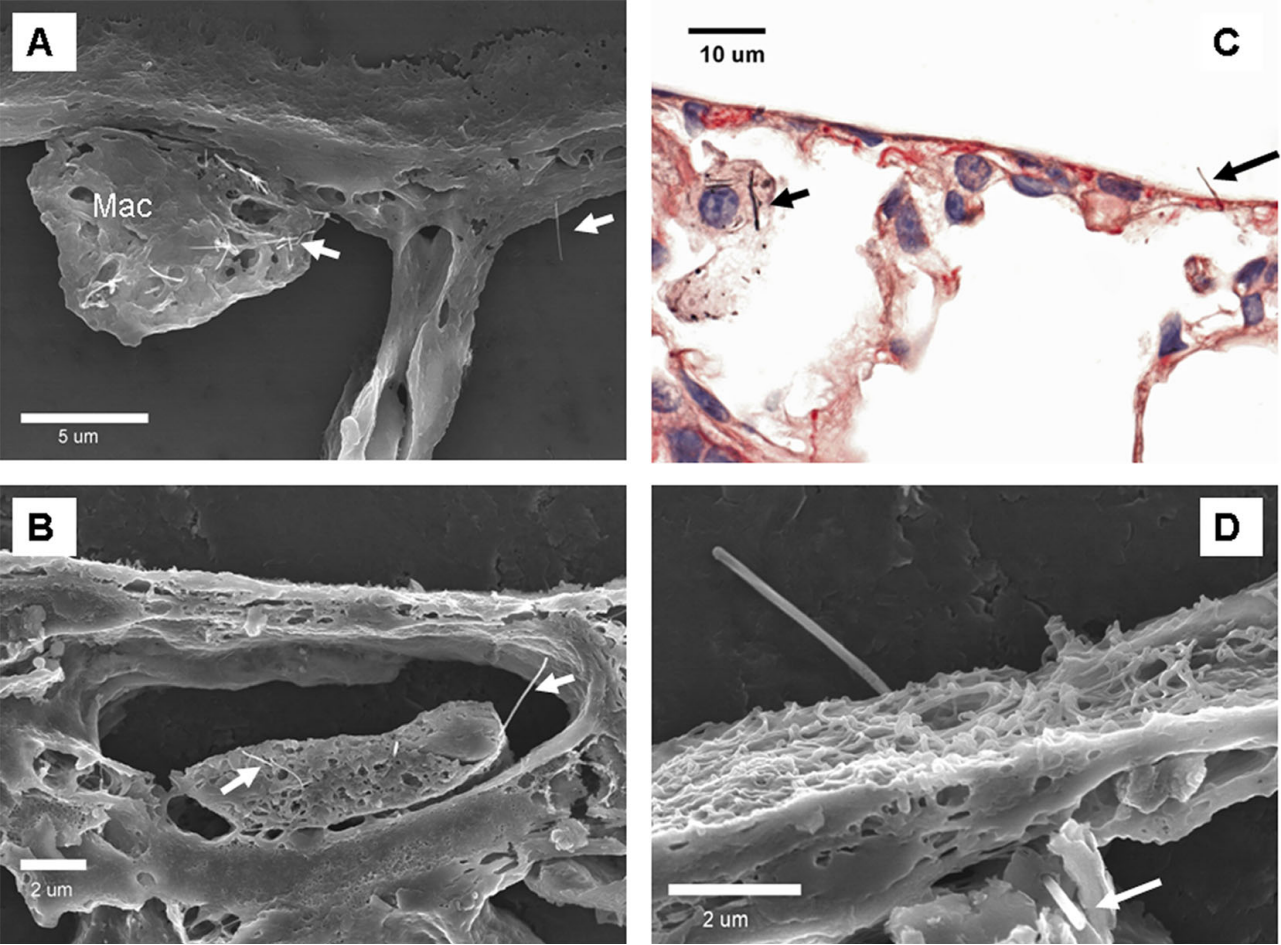
**Representative micrographs of MWCNT in subpleural tissues, visceral pleura and pleural space**. In all four panels, the visceral pleural surface runs along the top of each micrograph. The FESEM image in A shows a MWCNT loaded alveolar macrophage in an alveolus immediately beneath the visceral pleura surface. The right side of the image shows a single MWCNT fiber penetrating the alveolar epithelium into the subpleural tissues (80 μg dose, 28 day post-aspiration). Figure 7B shows a dilated subpleural lymphatic vessel which contains a mononuclear inflammatory cell that is penetrated by several MWCNT fibers (80 μg dose, 56 day post-aspiration). A MWCNT penetrating the visceral pleura is shown in the light micrograph of 7C with a MWCNT-loaded alveolar macrophage visible in the left side of the micrograph (80 μg dose, 28 day post-aspiration). A single MWCNT penetrating from the subpleural tissue through the visceral pleura into the pleural space is shown in the FESEM image of 7 D (80 μg dose, 56 day post-aspiration).

Figure 7 provides micrographic examples of the various types of MWCNT penetrations observed in the visceral pleura and immediate adjacent subpleural tissues. The visceral pleural surface runs along the top in each micrograph with a clear visualization of the abundant microvilli of mesothelial cells in Figure 7D. The micrograph in Figure 7A (80 μg, 28 days) shows both a MWCNT-loaded alveolar macrophage and a single MWCNT fiber penetrating into the subpleural tissues. Subpleural tissue and visceral pleural penetrations were frequently observed in areas which also had one or more MWCNT-loaded alveolar macrophages such as shown in this figure. There was also significant subpleural involvement and associated MWCNT penetrations of the subpleural lymphatics as illustrated by Figure 7B (80 μg, 56 days) which shows a dilated subpleural lymphatic with several MWCNT penetrating a mononuclear inflammatory cell within the lymphatic vessel. The light micrograph of Figure 7C (80 μg, 28 days) captures a MWCNT penetration into the intrapleural space (right arrow). An alveolar macrophage loaded with MWCNTs is also in the field of view (left arrow). The FESEM of Figure 7D (80 μg, 56 days) shows a MWCNT penetrating the mesothelial surface of the visceral pleura, through the mesothelial cell layer with the fiber ending in the alveolar region. The microvilli of the pleural mesothelial cells which are particularly elongated in the visceral pleura are evident in this view [19].

**Figure 8:**
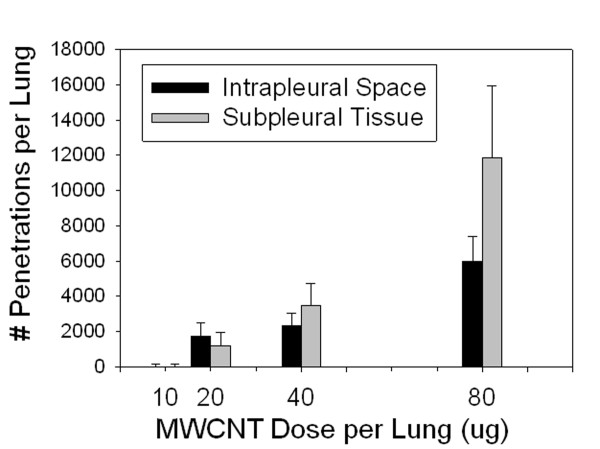
**Effect of MWCNT dose on MWCNT fiber penetrations into subpleural tissue and intrapleural space 56 days after aspiration**. This figure shows the number per lung for fibers which penetrated into the subpleural tissue and for fibers which penetrated into the intrapleural space. As indicated by the asterisks, both the number of subpleural tissue and intrapleural space penetrations at 80 μg were different from the corresponding subpleural tissue and intrapleural space penetrations at doses of 10, 20 and 40 μg. (Mean ± SE, N = 8).

Measurements of the number of MWCNT fiber penetrations into subpleural tissue and intrapleural space at different doses of MWCNT are given in Figure 8. No intercepts of subpleural tissue by MWCNTs were detected at the lowest dose of 10 μg while at the higher dose of 80 μg there were 11,900 penetrations of the subpleural tissue per lung and 5,980 penetrations into the intrapleural space of the lungs.

**Figure 9:**
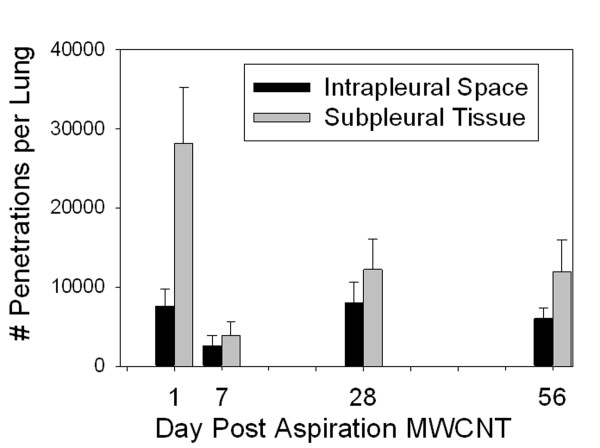
**Time course of MWCNT penetrations into the subpleural tissue and intrapleural space**. There was a significant number of penetrations into the subpleural tissue and intrapleural space with an initial spike at one day after aspiration with some initial clearance evident by the decline at 7 days. However the number of penetrations in both spaces significantly increased after day 7 and was still elevated 56 days after the initial aspiration. Data from animals given a single aspiration dose of 80 μg. As indicated by the asterisk, the number of subpleural tissue penetrations at 1 day was significantly different from the number of penetrations at 7, 28 and 56 days. (Mean ± SE, N = 8).

The time course of fiber penetrations into the subpleural tissue and into the pleural surface is given in Figure 9. One day post aspiration there were substantial fiber penetrations with some initial clearance, evident by the decline at 7 days. By 7 days the number of subpleural tissue penetrations declined to approximately 20% of the subpleural tissue penetrations at one day. Following the initial decline between day 1 and 7, the number of subpleural and intrapleural space penetrations increased at the longer time points of 28 and 56 days.

## Discussion

Animal studies of MWCNT toxicity have generally found evidence for an acute inflammatory phase lasting approximately 7 days with chronic and persistent Type II cell hyperplasia, progressive fibrotic thickening of the alveolar wall and a varying size and extent of granulomatous inflammation depending on how well dispersed the MWCNT were during exposure [10,20-23]. These chronic effects have been shown to persist out to 91 days and are qualitatively similar to those observed for SWCNT [24].

Given the similarity in time-course and types of responses to SWCNT versus MWCNT, it might be assumed that the two CNTs react similarly in tissues. However, examination of lung tissues, particularly with high resolution FESEM or TEM, demonstrates that MWCNT interactions with lung tissue are remarkably distinct from those of SWCNT. When highly dispersed into submicron structures SWCNT do not form granulomatous lesions but instead are incorporated into the interstitium in a diffuse distribution throughout the lungs. Indeed the dispersion is so extensive that individual nanotubes are difficult to identify without being tagged with an easily detectable label such as colloidal gold [16]. In lungs exposed to MWCNT in our study, the individual fibers are generally easy to identify and can be followed as they penetrate into and/or through cells and tissue layers (Figure 7.). In SWCNT exposed lungs only a small percentage of SWCNT structures were found in alveolar macrophages [16]. In contrast, alveolar macrophages in MWCNT-exposed lungs contained numerous MWCNT. Many of the MWCNT penetrations traverse completely through the cell and/or cell nucleus [10]. The fiber load within alveolar macrophages was approximately 62% of the total lung burden at day 1 post-exposure.

The penetrations by MWCNT were not limited to phagocytic cells. As shown by the results of this study, there are high frequency penetrations in both the alveolar epithelium and visceral pleura. The alveolar epithelial cell penetrations of Figure 5 show in excess of 20 million penetrations throughout the lungs at a dose of 40 μg or greater.

It was originally expected that the penetrations of the subpleural tissue would follow a similar time and dose response as the alveolar epithelial cell penetrations. This was based on the hypothesis that both involved a similar mechanism by which the alveolar epithelium was penetrated with the only difference being that the subpleural tissue was by definition alveolar epithelium immediately below the visceral pleural. The results of Figure 5 and Figure 8 demonstrate that these two epithelial boundaries are not penetrated by MWCNT in the same time versus dose response. There were significant numbers of subpleural and intrapleural penetrations at 20, 40 and 80 μg doses per lung (Figure 8). However, there were essentially none observed at the lowest dose of 10 μg used in the study. At the same time the alveolar epithelial cell penetrations at the lowest dose were 20% of those observed at 80 μg (Figure 5).

It is possible that macrophage clearance of fibers at the 10 μg dose was sufficient to protect the most distal lung regions such as the pleura. However, it seems unlikely that this is the case given the high levels of epithelial penetrations elsewhere at this dose. The differences in transport to the two regions indicate that MWCNT penetration of the visceral pleura is distinct from MWCNT penetration of the alveolar epithelium that occurs elsewhere in the lungs. It has been suggested that dilated subpleural lymphatics may attract macrophage containing phagocytized MWCNT to the subpleural tissue [10].

Donaldson et al. [9] argue that fiber length is critical to the development of pleural pathology, since short fibers should be cleared from the intrapleural space via ducts in the parietal pleura which drain into the lymphatics. The length of MWCNT used in the present study is 3.9 μm [10]. Given this length, it would be expected that MWCNT would be cleared from the intrapleural space via these ducts [9]. Such clearance was observed from 1 to 7 days post aspiration. However, intrapleural levels of MWCNT rose once again at 28 days and remained constant through 56 days post aspiration. It is possible that the lung burden of MWCNT acts as a reservoir to replenish MWCNT in the intrapleural space. It is also possible that the 3.9 μm fibers could reach a level within the intrapleural space at which ducts begin to be clogged. NIOSH is currently conducting a 12 day inhalaton study for MWCNT with an evaluation period as long as 1 year post-exposure which should elucidate issues concerning long-term kinetics of penetration vs clearance of MWCNT from the intrapleural space.

In this study we have demonstrated rapid and direct transport of MWCNT to the visceral pleura (Figures 2, 7, 8 and 9). Ryman-Rasmussen et al. [12] and our own laboratory group [10,11] have reported deposition of MWNCT in the subpleural tissue after inhalation or aspiration exposure. However, to our knowledge there are no available data on nanomaterial penetrations of pleura. Transport of man-made mineral fibers to the pleura have been reported. Gelzleichter et al. [25] found transport of 0.09% of the lung burden to the rat pleura following a 12 day exposure at 83 mg/m^3 ^to refractory ceramic fiber (RCF-1). Bermundez et al. [18] exposed rats or hamsters for 12 weeks to man-made vitreous fibers (85 mg/m^3^). They reported 5% and 0.5% of the total lung fiber burden (between 1 and 8 μm in length) was transported to the pleural surface of the hamster and rat, respectively. While not directly comparable due to species differences and the obvious larger size of the synthetic fiber, our result demonstrating a 0.6% transport 1 day following the aspiration of 80 μg of MWCNT is within the wide range reported by these studies (Figure 2).

In the case of human exposures, fiber burdens have been evaluated in workers from a number of different occupational exposures settings. As indicated by Miserocci et al. [26], evaluation of human lung tissue from individuals exposed to asbestos is difficult to conduct due to the decades of life over which asbestos translocation occurs. Typically, human studies involve detailed measurements of the length and types of asbestos fibers in ashed or digested samples of lung parenchyma and pleura which are fibrotic in nature as these are more likely to contain asbestos fibers [27]. Suzuki and Kohyama [28] reported on a study of 13 insulators that fibrotic lung areas contained 64 x10^6 ^chrysotile fibers/g dry weight (wt) and 150 x10^6 ^amosite fibers/g dry wt while pleural tissue samples contained 46 x10^6 ^chrysotile fibers/g dry wt and 2.2 x10^6 ^amosite fibers/g dry wt. The transfer of asbestos fibers from the parenchyma to the intrapleural space can not be estimated without data on the relative masses of fibrotic lung areas and affected pleural sites. These masses would likely vary considerably depending on the exposure and stage of the disease. However, even assuming a ratio of 250 to 1 (the ratio of human alveolar surface area [17] to pleural surface area [29]) between mass of fibrotic lung area and mass of affected pleural sites would suggest a considerable transport to the pleural space. Experimental animal studies of chrysotile or amosite asbestos inhalation have had mixed conclusions on the extent to which asbestos fibers are transported to the pleura [30,31]. Even after 6 months of amosite exposure in hamsters, Hesterber et al [30] referred to the "occasional" penetration of the pleura by asbestos although all bronchioles in the lungs were affected at that advanced stage.

## Conclusions

In summary, the results of this study demonstrate that MWCNT penetrations of alveolar macrophages, the alveolar epithelium, and pleura are rapid, frequent and persistent. There is an acute phase clearance of MWCNT from the subpleural tissue which is likely due to macrophage and subpleural lymphatic activity, while acute clearance of MWCNT may involve flow of intrapleural fluid through stromal pores on the surface of the parietal pleura [9,27]. At 28 days post-exposure, an equilibrium is reached between this clearance and replenishment of intrapleural MWCNT from subpleural tissue. Available data on asbestos lung burden transport to the pleural space is incomplete but suggests that the significant and rapid transport of MWCNT to the intrapleural space may be distinctly different from the decades long process identified in asbestos exposed humans. In humans, the risk of mesothelioma development are the consequence of chronic exposure and chronic response to fibers. Results from the current acute study of a bolus aspiration of MWCNT are thus not directly comparable.

## Methods

### Animal

Male C57BL/6J mice (7 weeks old) were obtained from Jackson Laboratories (Bar Harbor, ME). Mice were housed one per cage in polycarbonate ventilated cages, which were provided HEPA-filtered air, with fluorescent lighting from 0700 to 1900 hours. Autoclaved Alpha-Dri virgin cellulose chips and hardwood Beta-chips were used as bedding. Mice were monitored to be free of endogenous viral pathogens, parasites, mycoplasms, Helicobacter and CAR Bacillus. Mice were maintained on Harlan Teklad Rodent Diet 7913 (Indianapolis, IN), and tap water was provided ad libitum. Animals were allowed to acclimate for at least 5 days before use. All animals used in this study were housed in an AAALAC-accredited, specific pathogen-free, environmentally controlled facility. All animal procedures were approved by the NIOSH ACUC.

### Carbon Nanotube Source

MWCNT used in this study were obtained from Mitsui & Company (XNRI MWNT-7, lot #05072001K28) and were fully characterized in a previous report [10]. Briefly, MWCNT trace metal contamination was 0.78% with sodium (41%) and iron (0.32%) being the major metal contaminants. MWCNT median length was 3.86 μm and count mean diameter of 49 ± 13.4 (S.D.)nm.

### Pharyngeal Aspiration of MWCNT

Suspensions of MWCNT for aspiration were prepared in a dispersion medium (DM) as reported previously [32]. Dispersion medium was made up of Ca^+2 ^and Mg^+2 ^phosphate-buffered saline (PBS), pH 7.4, supplemented with 5.5 mM D-glucose, 0.6 mg/ml mouse serum albumin and 0.01 mg/ml 1,2 dipalmitoyl-sn-glycero-3-phosphocholine (DPPC) DPPC was prepared fresh as a 10 mg/ml stock solution in absolute ethanol. DM has been shown to be an effective dispersing agent for multi-walled carbon nanotubes and does not elicit toxicity [32,33]. Recently published direct comparisons studies between inhalation and aspiration of SWCNT have further demonstrated the applicability of the aspiration technique for such studies [24].

Mice were anesthetized with isoflurane (Abbott Laboratories, North Chicago, IL) for pharyngeal aspiration. When fully anesthetized, the mouse was positioned with its back against a slant board and suspended by the incisor teeth using a rubber band. The mouth was opened, and the tongue gently pulled aside from the oral cavity. A 50 μl aliquot of sample was pipetted at the base of the tongue, and the tongue was restrained until at least 2 deep breaths were completed (but for not longer than 15 seconds). Following release of the tongue, the mouse was gently lifted off the board, placed on its left side, and monitored for recovery from anesthesia. In order to eliminate the possibility of incidental food aspiration, food was removed 4 hours prior to the procedure; water was removed 1 hour prior. This alternative to inhalation has been shown to produce a similar distribution of lung burden to that following inhalation and has been described in detail elsewhere [34]. Mice received either DM (vehicle control), 10, 20, 40 or 80 μg MWCNT.

At 1, 7, 28 and 56 days after aspiration, mice were euthanized by an overdose of pentobarbital (> 100 mg/kg body weight, i.p.) followed by transection of the abdominal aorta to provide exsanguination. Seven to 8 animals were studied at each time point. The lungs were fixed by intratracheal perfusion with 1 ml of 10% neutral buffered formalin after 30 minutes the lungs were removed from the chest cavity. Lungs were trimmed the same day, processed overnight in a tissue processor, and embedded in paraffin.

For morphometric studies, paraffin sections of the left lung (5 μm thick) were cut. A new region of the disposable knife blade was used to section each block in order to prevent potential cross-contamination that might result from MWCNT passage on the knife between sections. The sections were then deparaffinized and rehydrated with xylene-alcohol series to distilled water. To enhance the contrast between tissue and MWCNT, lung sections were stained with Sirius Red [35]. Sirius Red staining consisted of immersion of the slides in 0.1% Picrosirius solution (100 mg of Sirius Red F3BA in 100 ml of saturated aqueous picric acid, pH 2) for 1 - 2 hours followed by washing for 1 minute in 0.01 N HCl. Sections were then briefly counterstained in freshly filtered Mayer's hematoxylin for 2 minutes, dehydrated, and mounted with a coverslip. Additional sections were stained with hematoxylin and eosin for routine pathology assessment as previously reported [10].

### Field Emission Scanning Electron Microscopy

For scanning electron microscopy, sections of the lung were cut at 8 microns, placed on carbon planchets, deparaffinized and sputter coated. After coating, the specimens were examined with a Hitachi Model S-4800 Field Emission Scanning electron microscope (FESEM) at 5 to 20 kV. Use of thin sections from paraffin embedded tissue was found to be preferable to large, unevenly cut blocks because it provided a uniform thickness of organic material on the carbon planchet. The 8 micron sections were thick enough to convey three-dimensional information but were also less likely to charge or undergo shifts when examined at the high magnifications necessary to study nanomaterials.

### Lung Distribution of MWCNT

The distribution of MWCNT in the lungs was determined by counting the occurrence of MWCNT under an eyepiece point counting overlay using standard morphometric point counting methods [36] similar to those previously described for study of the distribution of SWCNT [16,36]. Point counting categories were subdivided into points over MWCNT in airway, points over MWCNT in the alveolar region, and points over MWCNT in the subpleural tissue region. Airway regions were defined as those containing airway tissue (airway epithelial cells-basement membrane and tissues of the broncho-vascular cuff), airway lumen, and associated blood vessels greater than 25 microns. Alveolar regions were those containing alveolar tissue and alveolar air space. The visceral pleura region included MWCNT in the subpleural tissue and MWCNT in the pleural surface. The subpleural tissue regions included the immediately subpleural alveolar interstitial-epithelium layer and subpleural lymphatics but did not include any portion of alveolar walls attaching to the pleura. Points in airway and alveolar regions were further subdivided into points over MWCNT that were in the airspace, points over MWCNT that were in tissue of the region, and points over MWCNT that were in macrophages.

To accomplish the counting, an eyepiece counting overlay consisting of 11 by 11 lines (121 total points for each throw of the overlay) was used with a 100× oil immersion objective. A grid pattern for throws of the counting overlay was used in order to insure a uniform sampling of the section which did not overweight interior points. The counting overlay throws of the eyepiece were positioned over the section at 12 uniformly spaced grid points in both × and Y co-ordinates. These 12 grid points were determined using the stage micrometer scale to measure the × and Y bounds of the section. Using the bounding rectangle of these co-ordinates a 3 by 4 grid was selected and the 12 intersections were used as the center point for each of the eyepiece counting overlay throws.

For each animal, three sections were counted and the counts for the airways, alveolar and subpleural tissue regions were summed. Each counting category was divided by this total and multiplied by 100 to express the results as a percentage of total lung burden.

### Alveolar Epithelial Penetrations

Penetrations of the alveolar epithelium by MWCNT fibers were determined by counting the number of fiber penetrations per unit area based on repeated throws of an eyepiece counting box overlay. To tabulate the counts for each section, 12 placements of the eyepiece counting box overlay were carried out using a grid placement system similar to that described for determination of the lung distribution of MWCNT. For each placement, a count was made of the MWCNT which penetrated the alveolar epithelium within the box. Total penetrations per unit area of lung section were divided by the volume of the counting box (area times thickness) to obtain the number of penetrations per unit volume which was then multiplied by the fixed lung volume to express the results as number of alveolar epithelial penetrations per lung. Section thickness was 5.14 μm. The section thickness was determined by taking micrometer measurements of a tissue block before and after cutting 2000 sections.. Two sections per animal were analyzed and the results averaged. Eight animals were analyzed per group.

### MWCNT Penetrations of the Subpleural Tissue and Intrapleural Space

For each lung section, the total number of penetrations of MWCNT into the subpleural tissue and into the intrapleural space was determined. Subpleural penetrations were defined as MWCNT fibers which penetrated from the alveolar air side of the pleura into the alveolar epithelium and interstitium/subpleural lymphatics immediately beneath the mesothelial cell layer of the visceral pleura. In the rudimentary visceral pleura of the normal mouse, this is essentially a 1 to 2 micron depth below the surface of the mesothelial cells forming the visceral pleura. Intrapleural penetrations were defined as MWCNT fibers which penetrated the surface of the visceral pleura. Occasionally fibers where sufficiently long to penetrate completely from the alveolar side through to the visceral pleural (see Figure 7 in Results). These cases were counted as both subpleural tissue and intrapleural penetrations.

The counting of penetrations was done by sweeping around the pleural perimeter of the section using a 100× oil immersion objective with constant refocusing as the complete perimeter of the visceral pleura (including interlobular septa) was scanned. These counts were divided by the area of the pleural surface in the cross section (perimeter length times the section thickness) to obtain the number of penetrations (subpleural and intrapleural) per unit of pleural surface area. The perimeter length of the pleura was determined from low magnification images digitized for each section and measured using ImageJ. To express the results as number of penetrations per lung, the penetrations per unit of pleural surface area were multiplied by the pleural surface area of the mouse lung (500 mm^3 ^,[29]). Correction for the potential over-estimation of counts due to the size dependent projection of the length of the fibers, Holmes effect[37], was not necessary as the counts were done of the intersections between the fiber and the surface. The area of intersection corresponds to diameter of the fiber which is ~51 nm. This is a constant factor between groups and is negligible relative to the section thickness used for counting. Two sections per animal were analyzed and the results averaged. Eight animals were analyzed per group.

### Statistical Analyses

Data were analyzed using analysis of variance (STATGRAF). Bartlett's test was used to test for homogeneity of variances between groups. Statistical differences were determined using one-way analysis of variance with significance set at p ≤ 0.05. When significant F values were obtained, individual means were compared to control using Duncan's multiple range test [38] and P < 0.05 was considered to be significant. Data are given as mean ± SE.

## Abbreviations

CNT: carbon nanotubes; DM: dispersion medium; DPPC: 1,2 dipalmitoyl-sn-glycero-3-phosphocholine; ED_50_: effective dose for 50 percent response; FESEM: field emission scanning electron microscope; MWCNT: multiwalled carbon nanotubes; PBS: phosphate-buffered saline; SWCNT: single-walled carbon nanotubes.

## Competing interests

The authors declare that they have no competing interests.

## Authors' contributions

RM conceived of the study, developed the morphometric methods, conducted the FESEM evaluation, analyzed the experimental results and drafted the manuscript. AH made the original observation of pleural penetrations by MWCNT and was involved in the planning and writing of the manuscript. JS performed the morphometric counting of the parenchyma and pleural samples and assisted in analysis of results. LW contributed to the experimental design and assisted in lung preparation. LB provided important information on sampling of the lungs for pleural study and conducted lung preparation for histopathology. DS-B assisted in the sampling design and operation of the FESEM studies. VC and DP contributed to the experimental design, acquisition of funding and writing of the manuscript. All authors read and approved the final manuscript.

## Disclaimer

The findings and conclusions in this report are those of the authors and do not necessarily represent the views of the National Institute for Occupational Safety and Health.
